# Impact of miR-29c-3p in the Nucleus Accumbens on Methamphetamine-Induced Behavioral Sensitization and Neuroplasticity-Related Proteins

**DOI:** 10.3390/ijms25020942

**Published:** 2024-01-11

**Authors:** Hang Su, Li Zhu, Linlan Su, Min Li, Rui Wang, Jie Zhu, Yanjiong Chen, Teng Chen

**Affiliations:** 1 College of Forensic Medicine, Xi’an Jiaotong University Health Science Center, Xi’an 710061, China; hangsu@xjtu.edu.cn (H.S.); zhu_li983@163.com (L.Z.); sulinlan@stu.xjtu.edu.cn (L.S.); lming1997@stu.xjtu.edu.cn (M.L.); wrwyy0867@stu.xjtu.edu.cn (R.W.); zhujie.xjtu@xjtu.edu.cn (J.Z.); 2 The Key Laboratory of Health Ministry for Forensic Science, Xi’an Jiaotong University, Xi’an 710061, China; 3 National Biosafety Evidence Foundation, Bio-Evidence Sciences Academy, Western China Science and Technology Innovation Harbor, Xi’an Jiaotong University, Xi’an 710115, China; 4 Department of Immunology and Pathogenic Biology, College of Basic Medicine, Xi’an Jiaotong University Health Science Center, Xi’an 710061, China; chenyanjiong@126.com

**Keywords:** iTRAQ, methamphetamine, miR-29c-3p, neuroplasticity, sensitization

## Abstract

Methamphetamine (METH) abuse inflicts both physical and psychological harm. While our previous research has established the regulatory role of miR-29c-3p in behavior sensitization, the underlying mechanisms and target genes remain incompletely understood. In this study, we employed the isobaric tags for relative and absolute quantitation (iTRAQ) technique in conjunction with Ingenuity pathway analysis (IPA) to probe the putative molecular mechanisms of METH sensitization through miR-29c-3p inhibition. Through a microinjection of AAV-anti-miR-29c-3p into the nucleus accumbens (NAc) of mice, we observed the attenuation of METH-induced locomotor effects. Subsequent iTRAQ analysis identified 70 differentially expressed proteins (DEPs), with 22 up-regulated potential target proteins identified through miR-29c-3p target gene prediction and IPA analysis. Our focus extended to the number of neuronal branches, the excitatory synapse count, and locomotion-related pathways. Notably, GPR37, NPC1, and IREB2 emerged as potential target molecules for miR-29c-3p regulation, suggesting their involvement in the modulation of METH sensitization. Quantitative PCR confirmed the METH-induced aberrant expression of *Gpr37*, *Npc1*, and *Ireb2* in the NAc of mice. Specifically, the over-expression of miR-29c-3p led to a significant reduction in the mRNA level of *Gpr37*, while the inhibition of miR-29c-3p resulted in a significant increase in the mRNA level of *Gpr37*, consistent with the regulatory principle of miRNAs modulating target gene expression. This suggests that miR-29c-3p potentially influences METH sensitization through its regulation of neuroplasticity. Our research indicates that miR-29c-3p plays a crucial role in regulating METH-induced sensitization, and it identified the potential molecular of miR-29c-3p in regulating METH-induced sensitization.

## 1. Introduction

Methamphetamine (METH) is a psychoactive substance that was first produced in 1893 and was widely used after World War II, making it one of the most harmful substances [1,2,3]. METH is a widely abused drug across the globe [4]. METH abuse can cause severe physical and psychological damage [5,6,7,8]. Studies have confirmed that METH exposure produces a significant euphoric effect by increasing extracellular levels of dopamine [9,10,11,12,13]. This enhanced reward effect of METH is believed to underlie its addictive properties. Research on the mechanisms of METH addiction has shown that the substance affects the brain’s reward system. The nucleus accumbens (NAc) is a critical brain region that mediates reward responses and is regarded as the principal striatal subregion impacted by drug-induced long-term molecular and morphological alterations [14,15]. Long-lasting drug-induced neuroadaptations in the NAc indicate that gene regulation network dysfunctions may underlie addiction. Numerous cellular and molecular alterations have been observed in the NAc after repeated psychostimulant exposure. These changes include the increased release of dopamine and glutamate in the NAc [16] and subsequent increased sensitivity of dopamine receptors [17,18], altered protein and non-coding RNA expression [19,20], and changes in neuronal and synaptic plasticity in the NAc [16,17,18], highlighting the significance of studying the molecular mechanisms underlying psychostimulant effects in detail.

MiRNAs are non-coding RNAs that can negatively regulate gene expression by targeting the 3′ untranslated region (3′-UTR) of certain mRNA sequences [21,22]. As post-transcriptional regulators, miRNAs are found in large amounts in the brain [19,23]. MiRNAs have been linked to neurological disorders and have been shown to control synaptic plasticity, neuronal development [19,24], and psychological diseases [25,26,27,28]. Our previous research demonstrated that modulating miR-29c-3p expression in the mouse NAc can modulate the behavioral sensitization induced by methamphetamine [29]. Changes in miR-29c-3p in NAc-mediated gene expression may be crucial in controlling drug addiction, but in comprehensive miR-29c-3p target gene profiling, the change remains unclear.

Here, we intend to identify the likely molecular mechanisms underlying the effect of miR-29c-3p on METH-induced behavioral sensitization. AAV vector inhibitor–tough decoy (TuD) 29c was microinjected into the NAc of mice to inhibit miR-29c-3p separately. Following the inhibition of miR-29c-3p expression, METH-induced sensitization and locomotor activity were reduced. We identified 70 differentially expressed proteins (DEPs) in NAc influenced by miR-29c-3p using isobaric tags for relative and absolute quantitation (iTRAQ). This was followed by bioinformatic analysis, and 22 up-regulated DEPs were identified to be the proteins of the miR-29c-3p target genes. IPA showed that GPR37, NPC1, and IREB2 (3 of the 22 up-regulated DEPs) were found to exhibit significant enrichment in behavior and nervous system development and function. Both the protein level and mRNA level of GPR37 were changed, consistent with the regulatory role of miRNAs modulating target genes and proteins. Our results demonstrated that *Gpr37* was the miR-29c-3p target gene, which may play an important role in regulating METH sensitization.

## 2. Results

### 2.1. Inhibition of miR-29c-3p in the NAc Diminished the Properties of METH

**Figure 1:**
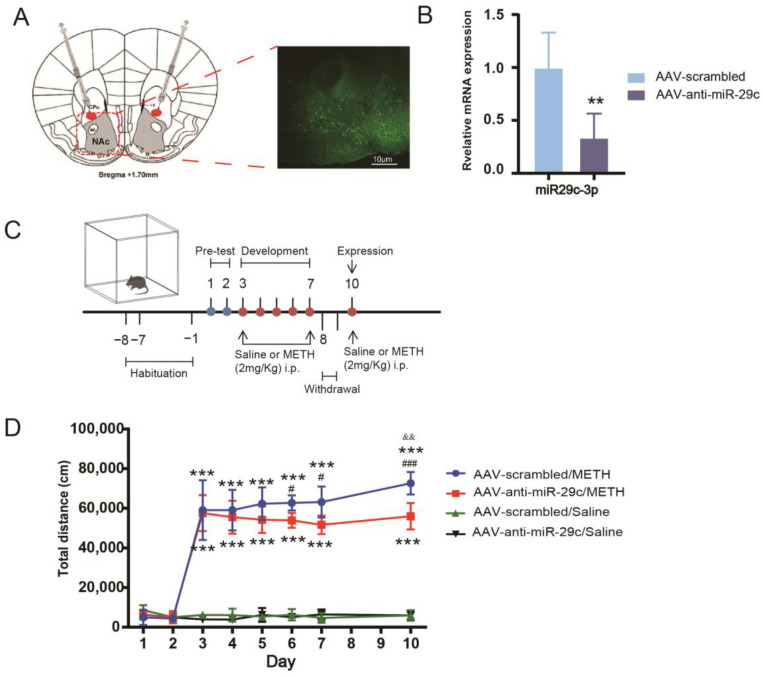
Inhibition of miR-29c-3p in the NAc attenuated METH-induced hyperlocomotion and behavior sensitization in mice. (**A**) Mice were microinjected with AAV-anti-miR-29c or AAV-scrambled on day 0. After 28 days, the location of AAV expression was observed using fluorescence microscopy to confirm eGFP expression (n = 3). eGFP was locally expressed in the NAc of mice, as visualized using fluorescence microscopy, ×100. (**B**) The expression of miR-29c-3p in the NAc of AAV-miR-29c mice. Expression levels were calculated relative to the levels of U6. All values are presented as the mean ± SD; t (10) = 3.944, ** *p* = 0.0028 (Student’s *t*-test); n = 6. (**C**) Timeline of the AAV-anti-miR-29c injection and behavioral test. (**D**) Mixed-design ANOVA followed by Bonferroni’s post hoc multiple comparison test was performed. Mixed-design ANOVA revealed significant main effects of AAV (F (1, 24) = 4.001, *p* = 0.057), METH (F (1, 24) = 1049.791, *p* = 0.000) and days (F (7, 168) = 214.580, *p* = 0.000), as well as the interaction of AAV × METH (F (1, 24) = 2.158, *p* = 0.155) and METH × days (F (7, 168) = 225.248, *p* = 0.000). *** *p* < 0.001, compared with the activity of the paired saline group at different time points and on day 10; && *p* < 0.01, compared with the activity of the same mice on day 3; # *p* < 0.05, ### *p* < 0.001, compared with the AAV-scrambled/METH group. All values are presented as the mean total distance ± SD, n = 6–8.

We investigated the impact of inhibiting miR-29c-3p expression in the NAc on lowering METH-induced behavioral sensitization. We employed AAV-anti-miR-29c or AAV-scrambled to inhibit the expression of miR-29c-3p in the NAc. We observed that eGFP expression mediated by AAV was restricted to the NAc (Figure 1A). The expression of miR-29c-3p was significantly decreased in the NAc of AAV-miR-29c mice (t (10) = 3.944, ** *p* = 0.0028) when METH was treated in the hyperlocomotion test (Figure 1C). Mice injected with AAV-anti-miR-29c or AAV-scrambled exhibited hyperlocomotion on each injection day (days 3–7 and day 10) compared with their paired saline group (*** *p* < 0.001, Figure 1D). The inhibition of miR-29c in the NAc significantly weakened the METH-induced locomotor activity of mice compared with the AAV-scramble/METH group, and the AAV-anti-miR-29c/METH group showed a reduced locomotor response to METH compared with AAV-scrambled/METH (days 6–7 and day 10, # *p* < 0.05, Figure 1D). Moreover, the AAV-anti-miR-29c/METH group of mice did not express a significant behavioral sensitization response to METH on day 10, and the AAV-scrambled/METH group of mice expressed significant behavioral sensitization (&& *p* < 0.01, Figure 1D). When saline was given, AAV-anti-miR-29c-injected mice displayed a level of locomotor activity that was not different from that of AAV-scrambled-injected mice from day 1 to day 10 (Figure 1D), demonstrating that the inhibition of miR-29c-3p expression did not alter the mice’s baseline locomotor activity.

### 2.2. Protein Expression Profile Involved in miR-29c-3p-Dependent METH-Induced Behavior Sensitization

**Figure 2:**
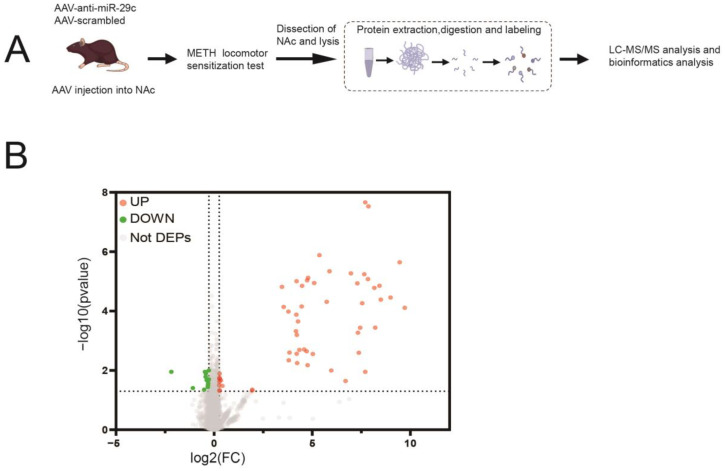
Analysis of the DEPs in AAV-miR-29c-regulated METH-induced sensitization using iTRAQ and LC-MS/MS. (**A**) The proteome analysis follows a systematic experimental procedure. (**B**) The volcano figure displays the changes in the log2 fold change (*x*-axis) and the −log10 *p* value (*y*-axis) for DEPs (AAV-anti-miR-29c/METH vs. AAV-scrambled/METH). The red dots indicate proteins that have been elevated, the green dots indicate proteins that have been down-regulated, and the gray dots indicate proteins that have not significantly changed. n = 3. DEPs, differentially expressed proteins; iTRAQ, isobaric tags for relative and absolute quantitation; IPA, Ingenuity pathway analysis; LC-MS/MS, liquid chromatography–mass spectrometry; NAc, nucleus accumbens.

We used iTRAQ and liquid chromatography–mass spectrometry (LC-MS/MS) to examine protein changes in the NAc of AAV-anti-miR-29c- and AAV-scrambled-injected mice that underwent the METH-induced hyperlocomotion test to identify targets of miR-29c-3p that were responsible for its effects on METH-induced behavior sensitization (Figure 2A). Proteins with a fold change ≥ 1.2 or ≤0.833 and a *p*-value < 0.05 were regarded as DEPs. A total of 70 DEPs were therefore found when AAV-anti-miR-29c/METH and AAV-scrambled/METH were compared with 13 down-regulated proteins and 57 up-regulated proteins, as shown in the volcano plot (Figure 2B).

**Figure 3:**
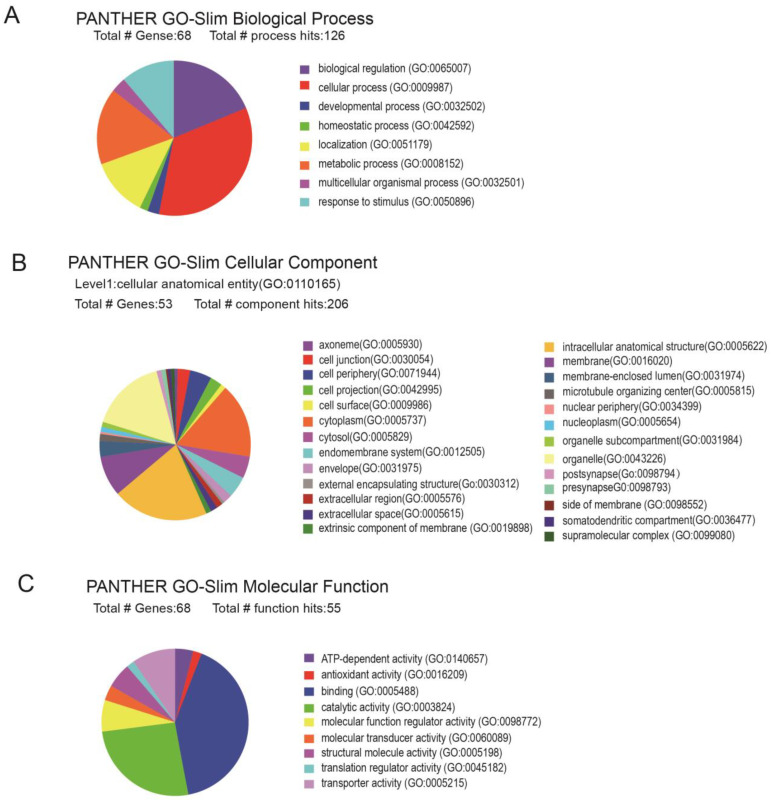
GO annotation analysis in AAV-miR-29c-regulated METH-induced sensitization. Mice GO annotation analysis of biological processes (**A**), cellular components (**B**), and molecular functions (**C**) of DEPs (AAV-anti-miR-29c/METH vs. AAV-scrambled/METH) by PANTHER GO are shown. Colored squares represent different cellular components.

Gene ontology (GO) functional enrichment was performed on 70 DEPs using the Panther database (http://www.pantherdb.org accessed on 8 November 2023) [30]. A total of 140 terms were obtained with a biological process; 84 terms were obtained with a cellular component; and 78 terms were obtained with a molecular function (Figure 3A–C). Following this, the aforementioned DEPs were extrapolated to the three primary application functions of GO. DEPs were enriched in the biological processes, including the cellular process, methylation, the G protein-coupled receptor signaling pathway, and apoptosis (Figure 3A). The results indicated that the DEPs were enriched in the synapses, cell junction, and membrane protein complexes in the cellular component (Figure 3B), in addition to the molecular function annotations of protein binding, iron binding, peptide binding, translation regulator activity, and transporter activity (Figure 3C).

**Figure 4:**
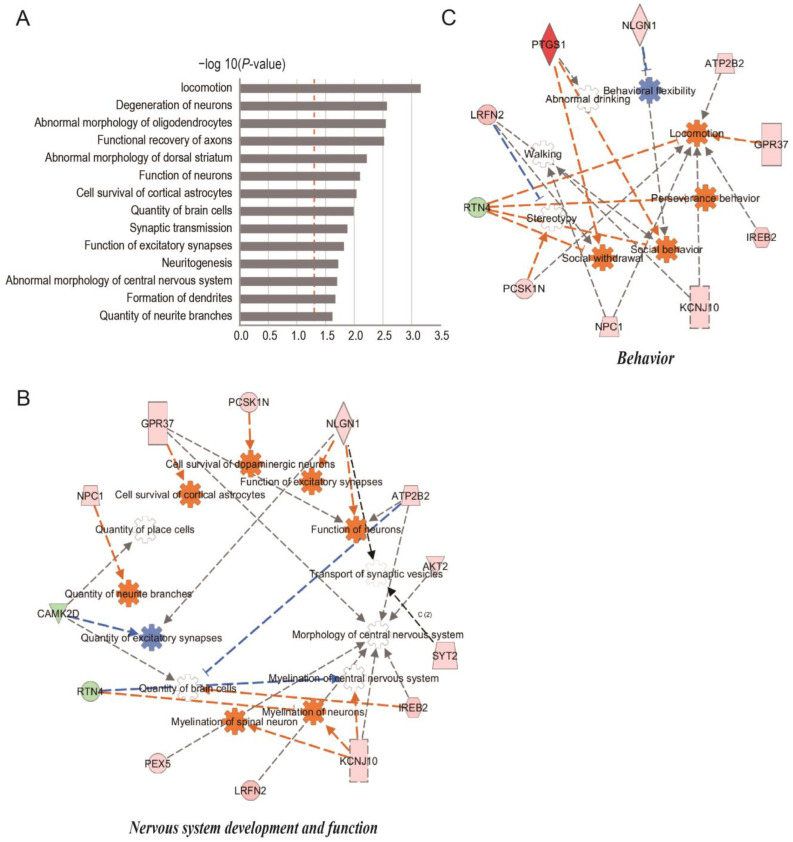
IPA analysis of the DEPs in AAV-miR-29c-regulated METH-induced sensitization. (**A**) The −log10 (*p* value) of the disease and functional terms of the DEPs identified by IPA are shown. The red dotted line indicates *p* = 0.05. (**B**,**C**) IPA analysis of the DEP interaction networks. The interaction networks of DEPs are annotated for (**B**) behavior and (**C**) nervous system development and function. The IPA database was utilized to evaluate the relationships and networks that were associated with diseases, functions, and DEPs. The functional class of each protein is represented by different shapes, while the expression variations of the protein are indicated by the colors of the shapes (red for up-regulated and green for down-regulated). For every function term, there is an orange shape for activation and a blue shape for inhibition. Differentiated arrows on dotted lines indicate the relationships between function terms and proteins. The line color—orange denotes activation, blue denotes inhibition, and grey denotes an effect that is not anticipated—indicates the degree of interaction. IPA, Ingenuity pathway analysis.

The DEPs were analyzed using Ingenuity pathway analysis (IPA) to gain insights into their molecular mechanisms through the annotation of diseases and biological functions and the identification of interaction networks. The processes linked to synaptic plasticity, neuronal morphology, neuronal function, and locomotion were significantly enriched in these 70 DEPs (Figure 4A and Supplementary Table S1). miR-29c-3p is capable of influencing molecular processes that are intrinsically linked to the regulation of METH-induced synaptic transmission, as demonstrated by these results. According to functional characterization, DEPs were enriched in behavior and nerve system development and function. Through the analysis of protein regulatory networks, 10 DEPs were identified to have substantial enrichment in behavior. These DEPs are primarily connected with locomotion, walking, and perseverance behavior. G protein-coupled receptor 37 (GPR37), iron-responsive element-binding protein 2 isoform X1 (IREB2), Niemann–Pick C1 protein isoform X1 (NPC1), proSAAS precursor (PCSK1N), plasma membrane calcium-transporting ATPase 2 isoform X7 (ATP2B2), ATP-sensitive inward rectifier potassium channel 10 (KCNJ10), and reticulon-4 isoform C (RTN4) were the proteins related to locomotion, as illustrated in Figure 4B. Thirteen DEPs were significantly enriched in nervous system development and function; these proteins were mainly connected to the function of neurons, quantity of neurite branches, and quantity of excitatory synapses; the cell survival of cortical astrocytes; and the cell survival of dopaminergic neurons, such as NPC1, neuroligin-1 (NLGN1), GPR37, IREB2, ATP2B2, and calmodulin-dependent protein kinase type II subunit delta isoform 1 (CAMK2D) (Figure 4C).

### 2.3. Comparison of miR-29c-3p Predicted Target Gene Interactions with DEPs

**Figure 5:**
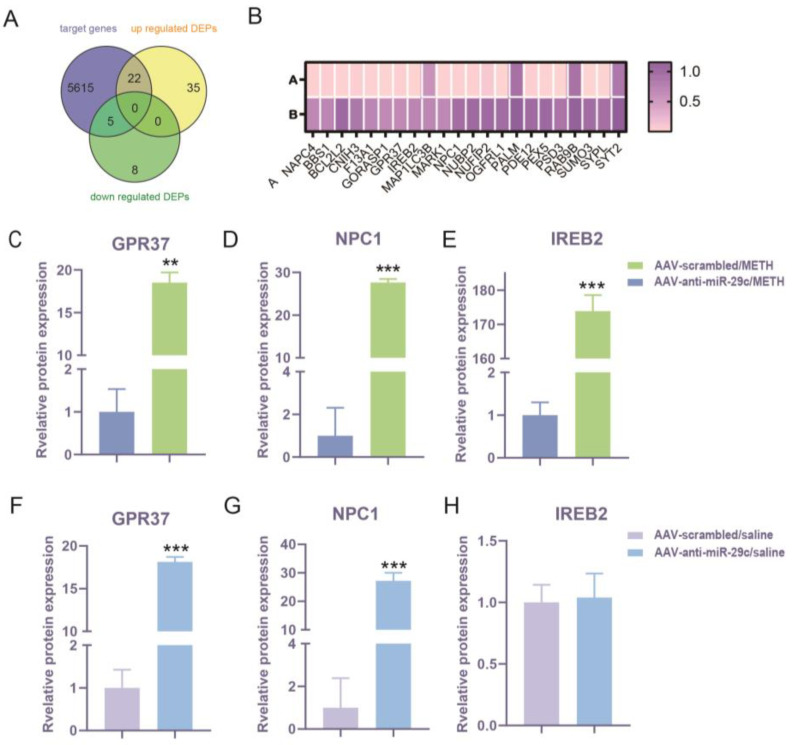
Predicted targets of miR-29c-3p were differentially expressed. (**A**) The Venn diagram shows the overlapping area of the predicted targets of miR-29c-3p, up-regulated DEPs, and down-regulated DEPs. A total of 5642 target genes were found. A total of 27 DEPs were predicted to be targets of miR-29c-3p, 22 DEPs were up-regulated, and 5 were down-regulated. (**B**) Heatmap of 22 predicted target DEPs of miR-29c-3p; A for the AAV-scrambled/METH group, B for the AAV-anti-miR-29c/METH group. Protein expression of GPR37 (**C**,**F**), NPC1(**D**,**G**), and IREB2 (**E**) was regulated by inhibition of miR-29c-3p in response to METH. (**C**–**E**) Comparison of AAV-anti-miR-29c/METH and AAV-scrambled/METH. (**F**–**H**) Comparison of AAV-anti-miR-29c/saline and AAV-scrambled/saline. Student’s *t*-test was used for the statistical analysis. AAV-anti-miR-29c/METH and AAV-scrambled/METH: GPR37: t(4) = 19.02, *p* = 0.0028; NPC1: t(4) = 29.76 *p* = 0.0001; IREB2: t(4) = 52.33, *p* = 0.0004. AAV-anti-miR-29c/saline and AAV-scrambled/saline: GPR37: t(4) = 34.25, *p* = 0.0009; NPC1: t(4) = 14.16 *p* = 0.0001; IREB2: t(4) = 0.2302, *p* = 0.8393.** *p* < 0.01, *** *p* < 0.001. All data are presented as the means ± SD, n = 3.

To enhance our understanding of the downstream target gene profile of miR-29c-3p that regulates METH-induced behavioral sensitization, we projected the target genes of miR-29c-3p using three prediction programs: miRPathDB (https://mpd.bioinf.uni-sb.de/ accessed on 29 September 2019), DIANA-micro T (https://www.hsls.pitt.edu accessed on 29 September 2019), and miRanda (http://www.microrna.org/ accessed on 29 September 2019) [31,32,33]. A total of 5642 genes were identified as possible miR-29c-3p targets. Upon interacting 70 DEPs with 5642 predicted target genes of mir-29c-3p, 27 DEPs were discovered to be encoded by miR-29c-3p target genes, 22 were up-regulated, and 5 were down-regulated (Figure 5A). A total of 22 up-regulated DEPs are shown in the heatmap (Figure 5B). IPA showed that GPR37, NPC1, and IREB2 (3 of the 22 up-regulated DEPs) were found to exhibit significant enrichment in behavior, nervous system development and function, as depicted in Figure 4B,C. Considering miRNAs can inhibit their target gene at the mRNA level and protein level, these findings suggests that miR-29c-3p may play a role in METH-induced behavior sensitization by affecting the quantity, density, and function of neurons, and locomotion. The high prevalence of GPR37, NPC1, and IREB2 underscores the ability of miR-29c-3p to control the structure and function of synaptic plasticity.

### 2.4. Effects of miR-29c-3p on the Expression Changes of GPR37, NPC1, and IREB2 in METH-Induced Behavioral Sensitization

GPR37, NPC1, and IREB2 may be involved in miR-29c-3p regulating METH-induced behavior sensitization. Thus, we determined whether miR-29c-3p regulates METH-induced behavior sensitization by targeting GPR37, NPC1, and IREB2. We found that the levels of GPR37 (*p* = 0.0028), NPC1 (*p* = 0.0001), and IREB2 (*p* = 0.0003) were significantly increased in the comparison of AAV-anti-miR-29c/METH with AAV-scrambled/METH (Figure 5C–E). The GPR37 (*p* = 0.0001), NPC1 (*p* = 0.0001), and protein levels were also significantly increased in the comparison of AAV-anti-miR-29c/saline and AAV-scrambled/saline (Figure 5F,G), but IREB2 (*p* = 0.8391) was not changed in these saline groups (Figure 5H).

**Figure 6:**
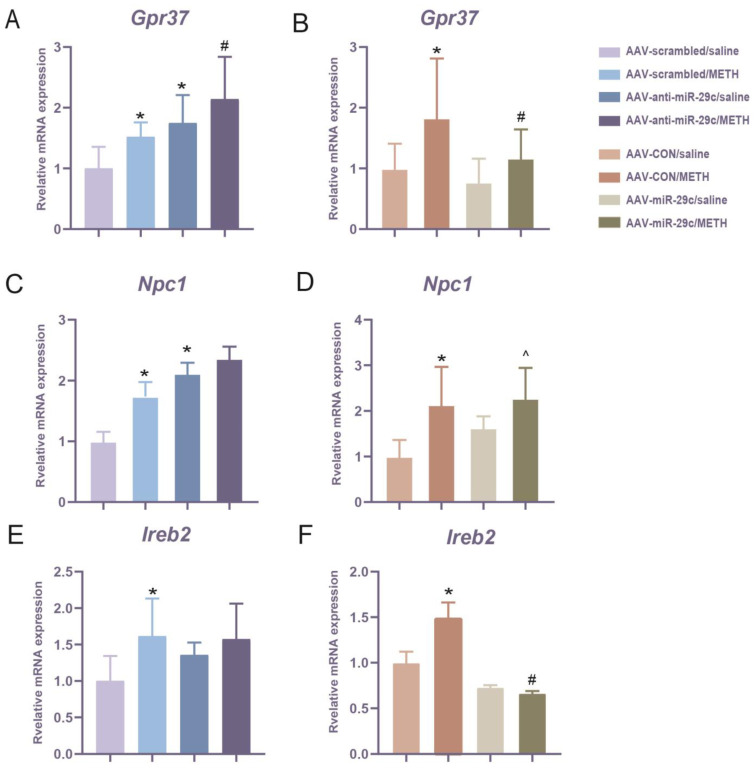
Changes in *Gpr37*, *Npc1*, and *Ireb2* expression were modulated by miR-29c-3p and METH. (**A**,**C**,**E**) *Gpr37*, *Npc1*, and *Ireb2* expression in the inhibition of miR-29c-3p. (**B**,**D**,**F**) MiR-29c-3p over-expressed in the NAc suppressed *Gpr37* and *Ireb2* expression, but not *Npc1*, in response to METH. The relative expression levels were calculated in reference to the levels of GAPDH. A two-way ANOVA followed by Bonferroni’s post hoc multiple comparison test was conducted. AAV-anti-miR-29c experiment: *Gpr37* fold change: METH: F(1, 25) = 6.383, *p* = 0.018; AAV: F(1, 26) = 14.078, *p* = 0.001; METH × AAV: F(1,25) = 0.126, *p* = 0.725. *Npc1*: METH: F(1, 26) = 5.392, *p* = 0.028; AAV: F(1, 26) = 17.052, *p* = 0.000; METH × AAV: F(1, 26) = 1.377, *p* = 0.251. *Ireb2*: METH: F(1, 23) = 6.638, *p* = 0.017; AAV: F(1, 23) = 0.953, *p* = 0.339; METH × AAV: F(1, 23) = 1.517, *p* = 0.23. AAV-miR-29c experiment: *Gpr37* fold change: METH: F(1, 36) =8.307, *p* = 0.007; AAV: F(1, 36) = 4.373, *p* = 0.044; METH × AAV: F(1, 36) = 1.06, *p* = 0.31. *Npc1*: METH: F(1, 36) =21.502, *p* = 0.000; AAV: F(1, 36) = 4.037, *p* = 0.052; METH × AAV: F(1, 36) = 1.604, *p* = 0.213. *Ireb2*: METH: F(1, 29) = 3.502, *p* = 0.073; AAV: F(1, 29) = 22.81, *p* = 0.000; METH × AAV: F(1, 29) = 5.877, *p* = 0.023; * *p* < 0.05, compared with AAV-scrambled/saline group or AAV-CON/saline group. # *p* < 0.05, compared with AAV-scrambled/METH group or AAV-CON/METH group; ^ *p* < 0.05, compared with AAV-scrambled/saline group. All values are presented as the mean ± SD. AAV-anti-miR-29c experiment: n = 5–9; AAV-miR-29c experiment: n = 8–12.

Then, we analyzed the mRNA level of *Gpr37*, *Npc1*, and *Ireb2* after miR-29c-3p regulation. METH treatment induced a significant increase in *Gpr37* expression in the AAV-scrambled/METH group and AAV-CON/METH group compared with the paired saline control group (* *p* < 0.05, Figure 6A,B). However, the inhibition of miR-29c-3p (AAV-anti-miR-29c/METH group) significantly promoted the METH-mediated increase in *Gpr37* expression, while the over-expression of the miR-29c-3p (AAV-miR-29c/METH) group attenuated *Gpr37* expression (# *p* < 0.05, Figure 6A,B). METH treatment induced a significant increase in *Npc1* expression in the AAV-scrambled/METH group and AAV-CON/METH group compared with the paired saline control group (* *p* < 0.05, Figure 6C,D). The *Npc1* expression level remained elevated in the AAV-miR-29c/METH group compared to the AAV-miR-29c/saline group (^ *p* < 0.05, Figure 6D). When miR-29c-3p was inhibited, *Npc1* expression was up-regulated compared with the AAV-scrambled/saline group (* *p* < 0.05), meanwhile *Npc1* expression in the AAV-anti-miR-29c/METH group showed a trend of up-regulation compared with the AAV-anti-miR-29c/saline group (* *p* = 0.061, Figure 6C). METH treatment induced a significant increase in *Ireb2* expression in the AAV-scrambled/METH group and AAV-CON/METH group compared with the paired saline control group (* *p* < 0.05, Figure 6E,F). However, the over-expression of the miR-29c-3p (AAV-miR-29c/METH) group attenuated *Ireb2* expression, while the AAV-anti-miR-29c/METH group did not promote a METH-mediated increase in *Ireb2* expression (# *p* < 0.05, Figure 6E,F).

### 2.5. Effects of METH on the Expression of Gpr37, Npc1, and Ireb2

To further associate the alterations in target gene expression with METH sensitization, we investigated whether *Gpr37*, *Npc1*, and *Ireb2* were modulated by METH in naïve unoperated mice; we found alterations in the NAc of *Gpr37*, *Npc1*, and *Ireb2* (METH group, * *p* < 0.05, ** *p* < 0.01). These findings demonstrated that repeated intermittent METH treatment increased *Gpr37*, *Npc1*, and *Ireb2* expression levels more than the saline treatment (Figure S1).

## 3. Discussion

Our current study demonstrates that inhibiting miR-29c-3p in NAc significantly reduces the hyperlocomotion activity induced by METH. iTRAQ protein expression profiles and IPA revealed that DEPs implicated in miR-29c-3p-regulated METH-induced sensitization were associated with altered nervous system development and function as well as behavioral modifications. Consistently, neuronal morphology and synaptic plasticity proteins, such as GPR37, NPC1, and IREB2, have been implicated in miR-29c-3p-regulated METH-induced behavioral sensitization.

MiR-29c-3p is an enriched miRNA in the brain that plays an important part in regulating neuroplasticity, involving learning and memory [34,35]. By suppressing mRNA expression or translation, miRNAs act as post-transcriptional regulators in various cellular processes. The relationships between miR-29c-3p and METH-induced behavioral alterations have been intermittently reported [29]. However, its underlying mechanisms and target gene profile remain incompletely understood. Thus, considering the bidirectional impact of miR-29c-3p manipulation on METH-induced behavior sensitization, the targets and molecular pathways that are most likely regulated should be identified.

In the case of AAV-anti-miR-29c/METH mice, we were able to identify DEP patterns in their NAc with the use of ITRAQ. Mice injected with METH and repressed with miR-29c-3p showed a significant increase of 81.43% (57/70) in DEPs. These elevated levels of DEPs may have a direct or indirect impact on the downstream signaling pathway, potentially leading to various alterations in neuronal function within the NAc. Indeed, the IPA analysis demonstrated high enrichment of DEPs in functions associated with neuronal functional changes in the NAc and neuroplasticity, including the postsynaptic membrane and postsynaptic specialization, as well as those involved in behavior or nervous system development and function. These results indicated that miR-29c-3p may affect METH sensitization through the regulation of neuronal morphology and synaptic plasticity.

Furthermore, evidence from reported studies showed that these specific up-regulated DEPs, GPR37, NPC1, and IREB2, played important roles in the regulation of neuronal morphology and synaptic plasticity. GPR37 is an orphan G protein-coupled receptor (GPCR) family member. GPR37 is widely expressed in the brain, mostly in neurons, and may play a role in the regulation of drug addiction [36]. Interestingly, we found that the inhibition of miR-29c in the NAc reduced METH-induced behavioral sensitization, while GPR37 was significantly elevated at both the protein level and the mRNA level after the inhibition of miR-29c. Further bioinformatics analysis revealed that *Gpr37* was involved in behavioral sensitization, and *Gpr37* was a potential target gene of miR-29c. Based on the above results, it can be concluded that the inhibition of miR-29c expression in NAc may reduce behavioral sensitization by increasing the expression of GPR37. The administration of METH treatment resulted in the notable up-regulation of *Ireb2* expression in both the AAV-scrambled/METH group and the AAV-CON/METH group, when compared to the corresponding saline control group. Nevertheless, the miR-29c-3p (AAV-miR-29c/METH) group attenuated up-regulated *Ireb2*, while the AAV-anti-miR-29c/METH group did not promote a METH-mediated increase in *Ireb2* expression. When compared to the paired saline control group, METH administration significantly increased *Npc1* expression in the AAV-scrambled/METH and AAV-CON/METH groups. Curiously, the over-expression or inhibition of the miR-29c-3p group does not alter the METH-induced increase in Npc1 expression. Based on these observations, we speculated that *Gpr37* may be the target of miR-29c-3p and thus participates in the prevention of METH sensitization. *Npc1* and *Ireb2* are also involved in the behavioral sensitization of METH but may not be regulated by miR-29c-3p.

Evidence from reported studies shows that the specific up-regulated DEPs of GPR37 are hypothesized to be the targets of miR-29c-3p in the striatal microRNA-mRNA networks of neuroinflammation in multiple system atrophy [37]. Studies have shown that GPR37 plays a role in regulating the release of dopamine, a neurotransmitter involved in the brain’s reward system, in response to drugs of abuse such as cocaine, amphetamine, and alcohol [36,38,39]. Gpr37-KO mice exposed to cocaine and amphetamine did not develop conditioned place preference (CPP) or behavioral sensitization, and cocaine-induced increases in locomotion activity were reduced [39,40]. Interestingly, previous studies on GPR37 support our results. Our data show that *Gpr37* expression was elevated in the NAc of METH-induced behaviorally sensitized mice and that *Gpr37* expression was more up-regulated in METH behavioral sensitization attenuated by miR-29c-3p. Marazziti’s study used 20 mg/kg cocaine and 6 mg/kg amphetamines for acute treatment, and 20 mg/kg cocaine for chronic treatment, whereas our study used 2 mg/kg METH [39]. Whereas their research employed *Gpr37* KO mice, the systemic knockdown of *Gpr37* will result in adaptive modifications. These combined factors may account for our disparate results. Nevertheless, our observations indicate that this relationship may be more intricate. Other regulatory mechanisms or signaling pathways may interact with GPR37 during the inhibition of sensitization, resulting in increased GPR37 expression. In a previous study, *Npc1*^−/−^ mice had less locomotor activity than WT mice, and the administration of AAV9-*Npc1* increased their locomotor activity [41]. In addition, our findings indicate METH-induced locomotor activity and behavioral sensitization in mice with high NPC1 expression. In contrast, inhibiting miR-29c-3p suppressed the behavioral sensitization induced by METH, which was accompanied by increased NPC1 expression. These findings suggest that miR-29c-3p regulates the aberrant expression of NPC1, perhaps not directly, to influence the behavioral sensitization and altered locomotor activity induced by METH. In this study, METH-induced behavioral sensitization was accompanied by increased IREB2 expression in the NAc, whereas the nicotine addiction susceptibility gene *IREB2* was identified in a genome-wide association study (GWAS) of nicotine-addicted populations [42], and another study demonstrated that Ireb2^−/−^ mice had decreased locomotor activity [43]. These findings suggest that the aberrant expression of *Ireb2* is involved in behavioral sensitization. METH addiction and increased locomotor activity are closely related. In the present study, METH treatment was accompanied by an altered expression level of *Ireb2* but not the target by miR-29c-3p. Consequently, IREB2 is involved in METH-induced behavioral sensitization and altered locomotor activity.

Here, we found that *Gpr37* in the potential targets of miR-29c-3p, NPC1, and IREB2 was involved in structural and functional neuroplasticity. This research revealed that synaptic structural plasticity changed and more long-term depression (LTD) occurred [44,45]. A study of Gpr37-KO mice found that synaptic structural plasticity and synaptic plasticity molecules experience changes [44]. Furthermore, Gpr37-KO mice were associated with potentiate LTD in corticostriatal synapses [44,45]. NPC1 is highly abundant in sympathetic neuron axons, both presynaptic and postsynaptic [46,47]. Npc1^−/−^ mice exhibited abnormalities of neuronal dendrites, including the loss of synapses and deformation of dendrites [48]. Defects in NPC1 significantly affect synaptic transmission [49]. IREB2 is a central regulator of cellular iron homeostasis in vertebrates. Iron accumulation was observed in oligodendrocytes of the Ireb2^−/−^ mouse cortex and striatum [43]. In a TAG meta-analysis of population data, IREB2 was substantially correlated with tobacco use and enriched signaling pathways of multiple receptor-related synapses and synaptic transmission [50]. In this work, it was discovered that GPR37, NPC1, and IREB2 expression was enhanced in the NAc of mice with behavioral sensitization induced by METH. Combining this evidence, GPR37, NPC1, and IREB2 showed significant roles in regulating synaptic plasticity.

The differential alterations of GPR37 in miR-29c-3p-regulated METH sensitization suggest that miR-29c-3p may dysregulate synaptic plasticity in response to METH exposure.

One limitation of the current study is that the findings in this paper are based on male mice, but male and female mice respond differently to METH. Female individuals exhibit greater sensitivity to METH, and in rodents, females demonstrate higher locomotion activity than males [51,52,53]. Therefore, the results presented in this paper can only provide information on the response of males to METH. The impact of mir-29c on METH in female mice has not been explored in this study and warrants further investigation in future studies. Additionally, considering that behavioral sensitization could also represent a sign of positive symptoms associated with psychosis [54], our findings may be important for understanding the molecular mechanism of METH-induced psychosis, which warrants further investigation in future studies.

## 4. Materials and Methods

### 4.1. Animals

We utilized male C57BL/6 J mice obtained from Beijing Vital River Laboratory Animal Technology in Beijing, China. The mice weighed between 25 and 30 g and were employed in our research. The room had a 12 h light/dark cycle, a steady temperature (21–25 °C), and constant humidity (40–60%). Each cage housed four mice. The researchers provided the mice with unrestricted access to water and food. This study was authorized by the Xi’an Jiaotong University Ethics Committee and carried out in accordance with the Declaration of Helsinki’s ethical criteria.

### 4.2. Drugs

The methamphetamine hydrochloride, provided by the National Institute for the Control of Pharmaceutical and Biological Products in Beijing, China, was dissolved in 0.9% saline. It was then delivered at a dosage of 0.1 mL/10 g body weight.

### 4.3. Adeno-Associated Virus (AAV) eGFP Expression

AAV (serotype AAV2/8) was used for inhibitor-TuD (AAV-anti-miR-29c, GGCGCTAGGATCATCAACTAACCGATTTCAATCTAATGGTGCTACAAGTATTCTGGTCACAGAATACAACTAACCGATTTCAATCTAATGGTGCTACAAGATGATCCTAGCGCCACCTTTTTT). An AAV vector with a randomly arranged anti-miR-29c-3p sequence (referred to as AAV-scrambled) was employed as a negative control to assess any non-specific effects of targeting. The over-expression of miR-29c was achieved using AAV-miR-29c (TAGCACCATTTGAAAT CGGTTA), while AAV-CON was employed as the control for miR-29c over-expression. The virus was supplied by GeneChem and OBiO Technology, Shanghai, China. The final virus preparation was titrated using real-time PCR, and the titer of the viral vector was 1.57 × 10^13^ vg/mL.

### 4.4. Surgery with Stereotaxy

The mice were administered a combination of ketamine (100 mg/kg) and xylazine (10 mg/kg) for anesthesia, and then, positioned in stereotaxic apparatus (RWD, Shenzhen, China). The NAc was positioned at a 20° angle using an automated injection pump (KDS LegatoTM 130, USA) and a Hamilton microsyringe (Hamilton, Rneo, NV, USA) with the following coordinates: anterior/posterior (A/P): +1.6 mm; medial/lateral (M/L): ±2.6 mm from the bregma; and dorsal/ventral (D/V): −4.8 mm from the skull surface [29,55]. To allow the AAV complexes to diffuse, the microsyringe was left in situ for five minutes after the 500 nL infusion had finished. Subsequently, the skin incision was closed with sutures. To avoid infection, mice received intraperitoneal injections of penicillin (0.02 mg/kg) once a day for five days. The mice had free access to food and water and received normal care while being kept in a cage. Twenty-eight days following the AAV microinjection, the location of the microinjection was confirmed by observing eGFP via fluorescent microscopy (LeicaDM3000). After 28 days of AAV injection, when they were stably expressed, a behavioral test was conducted.

### 4.5. Animal Groups

For the animals used to confirm AAV-anti-miR-29c and AAV-scrambled based on eGFP expression, n = 3. To provide evidence that the administration of AAV-anti-miR-29c in the nucleus accumbens inhibited the expression of miR-29c, n = 6. For the behavior test, there were four groups: AAV-anti-29c/METH, AAV-scrambled/METH, AAV-anti-miR-29c/saline, and AAV-scrambled/saline, n = 6–8. For protein analysis (AAV-anti-29c/METH, AAV-scrambled/METH, AAV-anti-miR-29c/saline, and AAV-scrambled/saline), n = 3. For AAV-anti-miR-29c mRNA analysis (AAV-anti-29c/METH, AAV-scrambled/METH, AAV-anti-miR-29c/saline, and AAV-scrambled/saline), n = 5–9. For AAV-miR-29c mRNA analysis (AAV-29c/METH, AAV-CON/METH, AAV-miR-29c/saline and AAV-CON/saline), n = 8–12. For mRNA analysis of naïve mice (METH vs. saline), n = 8.

### 4.6. Open Field Test

Previous studies [29] were used to guide the method used for METH-induced behavioral sensitization and the amount chosen (2.0 mg/kg). The mice underwent a 7-day habituation period. The mice were subjected to a pre-test period of two days (days 1–2) in which they received a once-daily saline injection for METH-induced locomotor sensitization. The mice were subsequently given an i.p. injection of METH (2 mg/kg, METH group) or saline (saline group) once a day for five days (days 3–7, development phase), followed by two days without an injection (days 8–9, transfer phase). The mice were given a challenge injection of METH (2 mg/kg) or saline on day 10 (the expression phase). To ensure that the mice were acclimated to the experimental environment, they were brought in for one hour on each treatment day. Horizontal locomotor activity was measured in open-field apparatus of 43 cm × 43 cm × 43 cm. The measurements were taken 1 h before and after the injections using a SMART video tracking system (version 3.0; Panlab Technology for Bioresearch, Barcelona, Spain) during the pre-test, development, and expression phases. The mice were acclimated for 1 h in an open-field chamber prior to the administration of METH or saline at each stage, in order to minimize any increase in activity caused by novelty. To analyze the data, the mice’s locomotor activities for one hour after the injection were recorded. Twenty-four hours following the last injection, all of the mice were sacrificed, and the NAc was quickly removed for investigation.

### 4.7. RNA Extraction and Reverse Transcription

In order to confirm the presence of mRNA after exposure to METH, we collected total RNA from the NAc (both the core and shell included) of mice that underwent METH-induced sensitization. The extraction of total RNA was performed by employing a MiRNeasy Mini Kit (QIAGEN, Germantown, MD, USA). The concentration and purity of RNA were assessed using a Nanodrop spectrophotometer (Thermo Scientific, San Jose, CA, USA). An amount of 500 ng of total RNA was reverse-transcribed into 10 μL of cDNA using the PrimeScriptTM RT Master Mix (TaKaRa, Shiga, Japan) with the following settings: 37 °C for 15 min, 85 °C for 5 s, and 4 °C for 5 min.

### 4.8. Quantitative Real-Time PCR Analyses (qRT-PCR)

The expression levels of Gpr37, Npc1, and Ireb2 in the NAc of mice were measured after METH-induced sensitization. To carry out qRT-PCR, we used the SYBR Premix Ex TaqTM II (Perfect Real Time) kit from Takara Biomedical Technology (Beijing, China) and a Bio-Rad iQ5 system from Bio-Rad (Hercules, CA, USA) in the US.

The temperature was set to 95 °C for 30 s, 40 cycles of 95 °C for 5 s, and 55 °C for 30 s. Gapdh was the endogenous control. The 2^−∆∆Ct^ method [56] was used to calculate relative expression levels. The primers were as follows: *Gpr37*-F: GTGCATCGTGTGTCACAATTAC, *Gpr37*-R: GGAGGCAGAAGAAGATGATGAG; *Npc1*-F: CTTGTGTTGGGTGGCTATGA, *Npc1*-R: GAGCCTCTCTGTGTCATTGTAG; *Ireb2*-F: TGCAGTAAAACAGGGTGATTTG, *Ireb2*-R: TAAATTTCCTTGCCCGTAGAGT; miR-29c-3p-F: CGGTAGCACCATTTGAAATCGG, *Gapdh*-F: TGTGTCCGTCGTGGATCTGA, *Gapdh* -R: TTGCTGTTGAAGTCGCAGGAG.

### 4.9. Protein Extraction

The NAc tissues (both the core and shell included) were homogenized using a mixture of RIPA lysis buffer. The homogenates were centrifuged at 12,000× *g* for 20 min at 4 °C after being incubated for 60 min on ice. Using a BCA protein assay (Applygen Technologies Inc, Beijing, China), the protein concentrations in the supernatants were determined after their collection. For future usage, all of the protein homogenates were kept at −80 °C.

### 4.10. iTRAQ

#### 4.10.1. Protein Labeling

The iTRAQ labeling process involved the use of 200 μg of protein per sample as a measure. A total of six samples, divided equally between the control and treatment groups, were utilized. The iTRAQ^®^ Reagents-8plex manufacturer’s procedure (AB Sciex Inc., Framingham, MA, USA; Sciex iChemistry^®^ Product Number 4390812) was followed for labeling proteins after they were digested. The iTRAQ^TM^ tags comprised three groups: a reporter, a peptide-reactive group (PRG), and a balance group. The peptides were incubated with various isobaric tags for two hours at room temperature. iTRAQ 8–113, iTRAQ 8–114, and iTRAQ 8–115 were assigned to the control samples (AAV-scrambled/METH), whereas iTRAQ 8–116, iTRAQ 8–117, and iTRAQ 8–118 were assigned to the treated samples (AAV-anti-miR-29c/METH). The proteins that were marked with labels were then broken down using MS/MS. Each peptide’s relative levels were proportionate to the relative intensities of the reporter ions obtained from the fragmentation process by the iTRAQ^TM^ reporter groups, which produced distinct ions.

#### 4.10.2. Strong Cation Exchange (SCX) Chromatography and LC-MS/MS Analysis

SCX chromatography was performed using Agilent 1260 Infinity high-performance liquid chromatography (HPLC) (Agilent Technologies, Santa Clara, CA, USA). A total of 40 fractions were gathered and combined into 20 fractions for LC-ESI-MS/MS analysis. The LC-MS/MS analysis was conducted using a DIONEX nano-UPLC system and a Q Exactive mass spectrometer (Thermo Scientific, San Jose, CA, USA). Following dissolution in Mobile Phase A, consisting of 99.9% H_2_O and 0.1% formic acid, the samples, approximately 1 μg in quantity, were introduced into the DIONEX nano-UPLC system for the purpose of peptide separation. Subsequently, steady loading of 2 to 35% Mobile Phase B (consisting of 0.1% formic acid and 99% ACN) was performed over a period of 45 min. This was followed by a flow rate of 300 nL/min for 1 min with 80% Mobile Phase B. A mass spectrometry study was conducted using a Q Exactive instrument equipped with an Orbitrap mass analyzer (Thermo Fisher Scientific, Carlsbad, CA, USA). The mass resolution was set at 70,000 FWHM at 400 *m*/*z*. Full scans were performed in the range of 350–1600 *m*/*z*. The MS/MS data obtained from each duty cycle were examined by analyzing the twenty-five most prominent precursor ions identified in a survey scan. The intense precursor ions were then detected using the Orbitrap analyzer, which provided a mass resolution of 17,500 FWHM at a *m*/*z* value of 400. All tandem mass spectra were generated using the higher-energy collision dissociation (HCD) method.

#### 4.10.3. Proteomic Analysis and Characterization of Protein Function

The LC-MS/MS data were searched against the mouse genome (Mus musculus, mmu) at the National Center for Biotechnology Information (NCBI) (https://www.ncbi.nlm.nih.gov/, accessed on 1 September 2018) using Proteome Discoverer software 1.4 (Thermo Scientific) and the Sequest algorithms. The settings of cysteine carbamidomethylation and iTRAQ modifications were set and fixed, while methionine oxidation was allowed to vary. A mass tolerance of 0.02 Da was observed for the fragment and 15 ppm for the precursor. Low-confidence peptides with a global false discovery rate (FDR) ≥ 1% were eliminated in the protein analysis using a target–decoy methodology. The iTRAQ quantitation analysis employed the most assured centroid approach, utilizing an integration window of 20 ppm. Protein quantification was performed exclusively on proteins that possessed a minimum of two distinct peptides. The median ratio from Mascot (Matrix Science, London, UK) was employed to adjust and standardize the quantitative protein ratios. In this investigation, only proteins with ratios (fold changes) greater than 1.2 or less than 0.833, and a *p*-value less than 0.05, were classified as differentially expressed proteins (DEPs).

### 4.11. IPA

The molecular function and regulatory mechanism of the DEPs were examined using the Ingenuity software (version: 2019 summer; apps.ingenuity.com, accessed on 1 June 2019) for the analysis, integration, and interpretation of “omics” data. To classify DEPs, an analysis was conducted on the annotation of biological functions and diseases, and also the identification of interaction networks. According to the IPA database, DEPs were categorized for disease and biological function into two categories: Behavior, nervous system development, and function. Interconnection networks were then examined and drawn between DEPs within these classifications. Statistical significance was set at *p* < 0.05 for all of these tests.

### 4.12. Statistical Analysis

Statistical analyses were performed using SPSS (version 25.0). All data are shown as mean *±* SD. The statistical analyses were performed using Student’s *t*-test, two-way ANOVA, and mixed-design ANOVA. For the behavioral test, the dependent variable was the locomotor activities (total distances). Mixed-design ANOVA followed by Bonferroni’s post hoc multiple comparisons test was used for analysis for the 8 days of locomotor activity testing, with days as a within-subject variable and treatments (AAV and METH) as a between-subject factor. Main and interaction levels were significant at *p* < 0.05. Protein and mRNA data were analyzed using Student’s *t*-test or two-way ANOVA followed by Bonferroni’s post hoc multiple comparisons test. The protein levels and the mRNA levels of the control group were set at 100%, and all data were normalized to the loading control. *p* < 0.05 was considered statistically significant.

## 5. Conclusions

In summary, our research indicates that miR-29c-3p potentially influences METH sensitization through the regulation of neuroplasticity. GPR37 within miR-29c-3p-regulated METH sensitization suggests a potential dysregulation of synaptic plasticity following METH exposure. This finding provides important clues for a deeper understanding of the role of miR-29c-3p in regulating METH sensitization.

## Data Availability

Data contained within the article.

## Supplementary Materials

The following supporting information can be downloaded at https://www.mdpi.com/article/10.3390/ijms25020942/s1.
