# Supplementary material for: Impact of miR-29c-3p in the Nucleus Accumbens on Methamphetamine-Induced Behavioral Sensitization and Neuroplasticity-Related Proteins
Source: Int J Mol Sci. 2024 Jan 11;25(2):942. doi: 10.3390/ijms25020942 (PMC10815255; doi:10.3390/ijms25020942)
Supplement: Supplementary file 1 [file ijms-25-00942-s001.zip › Figure s1.pdf]

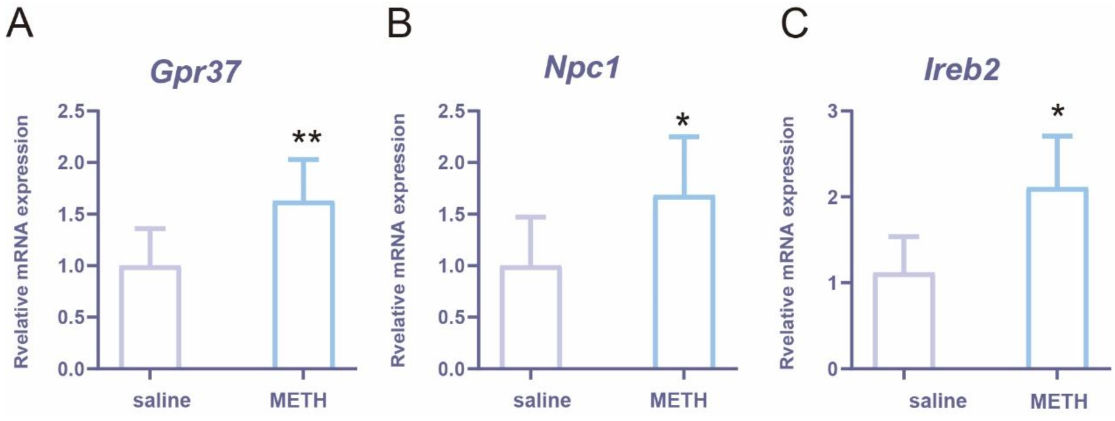

Figure S1. Changes in *Gpr37*, *Npc1*and *Ireb2* expression were modulated by METH. (A-C) qPCR showed differential expression of *Gpr37* (A), *Npc1*(B), and *Ireb2* (C) in the NAc of naive unoperated mice in response to METH. The relative expression levels were calculated relative to the levels of GAPDH. Student's t-test was used for the statistical analysis, *Gpr37* (A): $t(14)=3.304$, $P=0.0052$; *Npc1*(B): $t(14)=2.625$ $P=0.02$; *Ireb2* (C): $t(14)=2.317$, $P=0.0362$. * $P < 0.05$, *** $P < 0.01$; All data are presented as the means ± SD, n = 3. All values are presented as the mean ± SD; *$P < 0.05$, Student's t-test, n = 8.
